# Low Serum Cartonectin/CTRP3 Concentrations in Newly Diagnosed Type 2 Diabetes Mellitus: *In Vivo* Regulation of Cartonectin by Glucose

**DOI:** 10.1371/journal.pone.0112931

**Published:** 2014-11-19

**Authors:** Bo Ban, Bo Bai, Manman Zhang, Jiamiao Hu, Manjunath Ramanjaneya, Bee K. Tan, Jing Chen

**Affiliations:** 1 Department of Endocrine and Metabolic Diseases, Jining Medical College Affiliated Hospital, Jining Medical University, Jining, Shandong, P.R. of China; 2 Neurobiology Institute, Jining Medical University, Jining, Shandong, P.R. of China; 3 School of Medicine, Shandong University, Jinan, Shandong, P.R. of China; 4 Warwick Medical School, University of Warwick, Coventry, West Midlands, United Kingdom; 5 Department of Obstetrics and Gynaecology, Birmingham Heartlands and Solihull Hospitals, Heart of England NHS Foundation Trust, Birmingham, West Midlands, United Kingdom; Jilin University, China

## Abstract

**Objectives:**

Cartonectin is a novel adipokine of the C1q complement/TNF-related protein (CTRP) superfamily, with glucose lowering effects, anti-inflammatory and cardio-protective properties. We sought to investigate circulating cartonectin concentrations in subjects with type 2 diabetes mellitus (T2DM) as well as age and BMI matched control subjects. We also examined the effects of a 2 hour 75 g oral glucose tolerance test (OGTT) on serum cartonectin concentrations in T2DM subjects.

**Design:**

Cross-sectional study [newly diagnosed (first discovery, not on any treatments) T2DM (n = 47) and control (n = 63) subjects]. Serum cartonectin was measured by ELISA.

**Results:**

Serum cartonectin concentrations were significantly lower in patients with T2DM compared to controls (*P*<0.05). Furthermore, serum cartonectin was significantly negatively correlated with glucose and CRP, and significantly positively correlated with leptin, in all subjects (*n* = 110). When subjected to multiple regression analysis, none of these variables were predictive of serum cartonectin (*P*>0.05). There were no significant correlations in T2DM subjects (*n* = 47). In control subjects (*n* = 63), serum cartonectin was significantly negatively correlated with CRP, and significantly positively correlated with insulin, HOMA-IR and leptin. However, when subjected to multiple regression analysis, none of these variables were predictive of serum cartonectin (*P*>0.05). Finally, serum cartonectin concentrations were significantly lower in T2DM subjects after a 2 hour 75 g OGTT (*P*<0.01).

**Conclusions:**

Cartonectin may serve as a novel biomarker for the prediction and early diagnosis of T2DM patients. Furthermore, cartonectin and/or pharmacological agents that increase circulating cartonectin levels can represent a new therapeutic field in the treatment of T2DM patients. Further research is needed to clarify these points.

## Introduction

Obesity has now achieved pandemic proportions and left unchecked, leads to atherosclerosis by causing a repertoire of metabolic and cardiovascular perturbations such as type 2 diabetes mellitus (T2DM), dyslipidemia and hypertension [1]. Adipose tissue produces several hormones and cytokines termed ‘adipokines’ that have widespread effects on carbohydrate and lipid metabolism, which appear to play an important role in the pathogenesis of insulin resistance, diabetes, and atherosclerosis [2], [3]. Perturbations in adipokine concentrations have been reported in insulin resistant states such as gestational diabetes mellitus [4] and women with the Polycystic Ovary Syndrome (PCOS) [5].

Presently, there has been intense interest in the adipokines of the C1q complement/TNF-related protein (CTRP) superfamily. Foremost in this group is the adipokine adiponectin that circulates at high concentrations, and has insulin sensitizing, anti-inflammatory and anti-atherogenic properties [6]. Altered circulating adiponectin and adiponectin receptors levels have been described in patients with T2DM and insulin resistant states such as women with PCOS [7], [8]. Recently, CTRP3 (also known as cartonectin, cartducin, CORS-26) was reported as a novel adipokine, concentrations of which were lower in diet induced obese mice, with glucose lowering effects achieved by suppressing hepatic gluconeogenesis [9]. Cartonectin also decreases hepatic steatosis [10]. Cartonectin, similar to adiponectin, has anti-inflammatory properties [11]–[14]; cartonectin stimulates adipocyte adiponectin secretion [15]. Akiyama *et al*. had shown that cartonectin promotes proliferation and migration of endothelial cells and suggested that cartonectin may be a novel angiogenic factor in the formation of neo-intima following angioplasty [16]. Furthermore, Maeda *et al*. had demonstrated a biological role of cartonectin in promoting vascular smooth muscle cell proliferation in blood vessel walls after injury [17]. Moreover, Yi *et al*. had shown that cartonectin is a cardio-protective adipokine [18]; this could be via its protective effects on bone marrow derived mesenchymal stem cells, crucial for tissue regeneration in the ischemic myocardium [19]. More recently, Zhou *et al*. had reported that cartonectin promotes phosphate-induced smooth muscle vascular calcification [20].

Therefore, we measured circulating cartonectin concentrations in subjects with T2DM as well as age and BMI matched control subjects. We also examined the effects of a 2 hour 75 g oral glucose tolerance test (OGTT) on serum cartonectin concentrations.

## Materials and Methods

### Ethics

The study was approved by the medical ethics committee of the Affiliated Hospital of Jining Medical University, Shandong, China and written informed consent was obtained from all participants, in accordance with the guidelines in The Declaration of Helsinki 2000.

### Subjects

Forty-seven newly diagnosed T2DM (as per the 1999 World Health Organization criteria) [21] and sixty-three non-diabetic subjects participated in this study (Table 1). Newly diagnosed meant first discovery, and all T2DM subjects were not on any treatments at the time when their blood samples were obtained for this study. Exclusion criteria included a history of congestive heart failure, liver or kidney disease, malignancy, signs of inflammation, pregnancy and any drugs influencing body weight like corticosteroids or contraceptives. Subjects were patients of the Affiliated Hospital of Jining Medical University.

**Table 1:** Clinical, hormonal and metabolic features of study subjects.

Variable	T2DM (*n* = 47)	Controls (*n* = 63)	Significance
Sex (female/male)	22/25	28/35	
Age (year)	55.0 (41.0–60.0)	47.0 (40.0–54.0)	NS
BMI (kg/m^2^)	26.0 (22.9–28.4)	24.2 (22.0–26.4)	NS
SBP (mm Hg)	140.0 (125.0–150.0)	120.0 (108.0–127.0)	*P*<0.01
DBP (mm Hg)	85.0 (75.0–95.0)	75.0 (68.0–83.0)	*P*<0.01
TCH (mmol/L)	5.2 (4.4–5.9)	4.6 (4.1–5.1)	*P*<0.01
HDL-cholesterol (mmol/L)	1.4 (1.1–1.6)	1.4 (1.2–1.5)	NS
LDL-cholesterol (mmol/L)	3.0 (2.4–3.3)	2.7 (2.3–3.1)	NS
VLDL-cholesterol (mmol/L)	0.7 (0.4–1.2)	0.6 (0.4–0.7)	NS
Triglycerides (mmol/L)	1.6 (0.9–2.5)	1.0 (0.8–1.4)	*P*<0.01
TCH/HDL	3.8 (3.0–4.2)	3.5 (3.1–3.8)	*P*<0.05
TG/HDL	0.3 (0.2–0.5)	0.2 (0.2–0.3)	*P*<0.05
Glucose (mmol/L)	7.3 (6.7–9.3)	4.9 (4.6–5.3)	*P*<0.01
Insulin (pmol/L)	69.9 (32.6–92.6)	54.3 (36.7–72.0)	NS
HOMA-IR	3.1 (1.8–4.4)	1.7 (1.1–2.3)	*P*<0.01
HbA_1C_ (%)	7.3 (6.3–8.8)		
CRP (mg/l)	4.3 (2.2–8.7)	1.8 (0.8–2.6)	*P*<0.01
Leptin (ng/ml)	2.0 (1.0–5.4)	8.0 (3.1–12.0)	*P*<0.01
Adiponectin (µg/ml)	8.3 (6.1–21.4)	11.0 (8.4–16.2)	*P*<0.05
Cartonectin (ng/ml)	150.2 (72.9–288.7)	248.9 (115.0–321.2)	*P*<0.05

Data are median (interquartile range). Group comparison by Mann-Whitney *U* test.

BMI  =  body mass index; SBP  =  systolic blood pressure; DBP  =  diastolic blood pressure; TCH  =  total cholesterol; TG  =  triglycerides; DBP  =  diastolic blood pressure; NS  =  not significant.

After an overnight fast, blood samples were collected and immediately centrifuged. A 2 hour 75 g OGTT was also performed in subjects with T2DM. Serum was immediately aliquoted on ice and stored at −80°C. All patients underwent anthropometric measurements. Blood pressure was measured in a sitting position within a quiet and calm environment after a rest of at least 5 minutes. The average of three measurements was obtained.

The primary aim of our study was to investigate serum cartonectin concentrations in newly diagnosed (first discovery) subjects with T2DM.

### Biochemical and hormonal analysis

Assays for glucose, HbA1c (only in T2DM patients), total cholesterol (TCH), HDL-cholesterol, LDL-cholesterol, VLDL-cholesterol and triglycerides (TG) [Hitachi 7600 biochemical automatic analyzer] as well as for insulin (Roche electrochemiluminescence analyzer) were performed. The estimate of insulin resistance by HOMA-IR score was calculated as I_o_×G_o_/22.5, where I_o_ is the fasting insulin and G_o_ is the fasting glucose, as described by Matthews *et al*. [22]. The TCH/HDL and TG/HDL ratios were calculated as indices of ischaemic heart disease mortality and morbidity [23], [24]. CRP concentrations in sera were measured using a commercially available ELISA kit (Aviscera, Santa Clara, USA) according to manufacturer's protocol, with an intra-assay coefficient of variation of less than 6%. Leptin concentrations in sera were measured using a commercially available ELISA kit (Aviscera, Santa Clara, USA) according to manufacturer's protocol, with an intra-assay coefficient of variation of less than 8%. Adiponectin concentrations in sera were measured using a commercially available ELISA kit (Biovision, Milpitas, USA) according to manufacturer's protocol, with an intra-assay coefficient of variation of less than 8%. Cartonectin concentrations in sera were measured using a commercially available ELISA kit (USCN Life Science, Inc., Wuhan, China) according to manufacturer's protocol, with an intra-assay coefficient of variation of less than 10%.

### Statistical analysis

Data were analysed by Mann-Whitney *U* test and Wilcoxon matched pairs test. Data are medians (interquartile range). Spearman Rank correlation was used for calculation of associations between variables. Subsequently, if individual bivariate correlations achieved statistical significance, variables were entered into a linear regression model and multiple regression analysis was performed. All statistical analyses were performed using SPSS version 22.0 (SPSS, Inc.). *P*<0.05 was considered significant.

## Results

Table 1 shows the anthropometric, biochemical and hormonal parameters in all subjects. Systolic Blood Pressure (SBP), Diastolic Blood Pressure (DBP), TCH, TG, TCH/HDL ratio, TG/HDL ratio, glucose, HOMA-IR and CRP were significantly higher, whereas leptin and adiponectin were significantly lower in T2DM subjects.

Serum cartonectin concentrations were significantly lower in patients with T2DM compared to controls [150.2 (72.9–288.7) *vs.* 248.9 (115.0–321.2) ng/ml; *P*<0.05: Table 1].

Furthermore, women had higher serum adiponectin concentrations [10.6 (7.0–24.8) *vs.* 7.2 (5.4–12.4)µg/ml; *P*<0.05] compared to men; however, there were no significant difference with respect to serum cartonectin concentrations [224.1 (98.8–321.2) *vs.* 188.9 (100.7–304.0) ng/ml; *P*>0.05].

### Effects of a 2 hour 75 g OGTT in T2DM subjects on serum cartonectin concentrations

**Figure 1:**
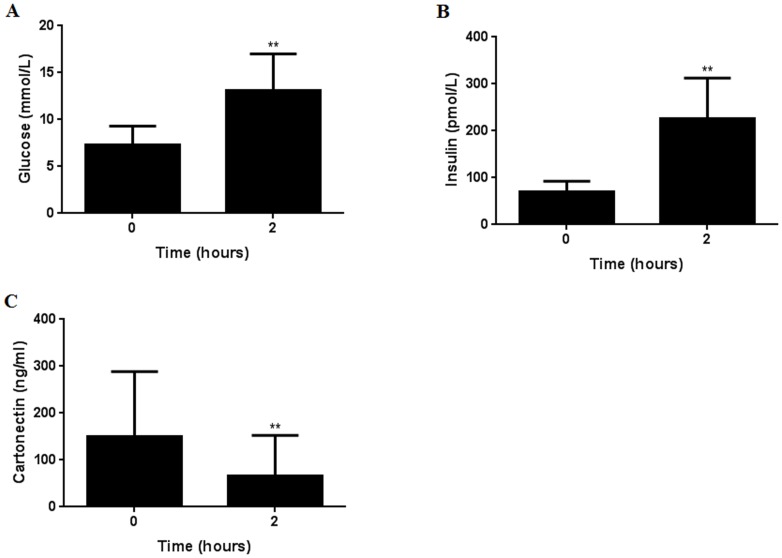
Effects of a 2 hour 75 g OGTT on serum glucose (A), insulin (B) and cartonectin (C) concentrations in T2DM subjects (*n* = 47). Group comparison by Wilcoxon matched pairs test. **P<0·01.

Glucose and insulin concentrations were significantly higher in T2DM subjects after a 2 hour 75 g OGTT (Figure1A: ^**^ *P*<0.01 and Figure1B: ^**^ *P*<0.01, respectively).

Conversely, serum cartonectin concentrations were significantly lower in T2DM subjects after a 2 hour 75 g OGTT (Figure1C: ^**^ *P*<0.01).

### Correlation of cartonectin with covariates

Spearman Rank analysis showed that serum cartonectin was significantly negatively correlated with glucose and CRP, and significantly positively correlated with leptin, in all subjects (*n* = 110) [Table 2]. When subjected to multiple regression analysis, none of these variables were predictive of serum cartonectin (*P*>0.05). There were no significant correlations in T2DM subjects (*n* = 47) [Table 2]. In control subjects (*n* = 63), serum cartonectin was significantly negatively correlated with CRP, and significantly positively correlated with insulin, HOMA-IR and leptin [Table 2]. However, when subjected to multiple regression analysis, none of these variables were predictive of serum cartonectin (*P*>0.05).

**Table 2:** Linear regression analysis of variables associated with Cartonectin.

Subjects	All (*n* = 110)		T2DM (*n* = 47)		Control (*n* = 63)	
Variable	*r*	*P*	*r*	*P*	*r*	*P*
Age (year)	0.008	0.930	0.204	0.168	−0.134	0.297
BMI (kg/m^2^)	−0.053	0.580	0.020	0.893	−0.013	0.918
SBP (mm Hg)	0.073	0.446	0.116	0.438	0.201	0.115
DBP (mm Hg)	0.049	0.612	0.079	0.597	0.207	0.103
TCH (mmol/L)	−0.158	0.099	−0.004	0.977	−0.169	0.185
HDL-cholesterol (mmol/L)	−0.031	0.747	−0.114	0.446	0.064	0.619
LDL-cholesterol (mmol/L)	−0.122	0.204	−0.047	0.753	−0.064	0.617
VLDL-cholesterol (mmol/L)	−0.027	0.778	0.223	0.132	−0.243	0.055
Triglycerides (mmol/L)	−0.014	0.888	0.012	0.936	0.145	0.255
TCH/HDL	−0.099	0.305	0.075	0.618	−0.136	0.289
TG/HDL	0.045	0.642	0.005	0.974	0.229	0.070
Glucose (mmol/L)	−0.201*	0.035	0.116	0.436	−0.194	0.127
Insulin (pmol/L)	0.159	0.096	0.092	0.537	0.388**	0.002
HOMA-IR	0.083	0.389	0.100	0.503	0.341**	0.006
HbA_1C_ (%)			−0.179	0.229		
CRP (mg/l)	−0.223*	0.019	0.064	0.667	−0.361**	0.004
Leptin (ng/ml)	0.274**	0.004	0.196	0.187	0.263*	0.037
Adiponectin (µg/ml)	−0.052	0.589	−0.080	0.594	−0.135	0.292

Spearman Rank correlation was used for calculation of associations between variables. If individual bivariate correlations achieved statistical significance, multiple regression analysis with Cartonectin as dependent variable was performed to test the joint effect of these parameters on Cartonectin. Multiple regression analysis contained glucose, CRP and leptin (all subjects); insulin, HOMA-IR, CRP and leptin (control subjects). *P<0.05; **P<0.01.

## Discussion

We report for the first time that serum cartonectin concentrations are significantly lower in newly diagnosed (first discovery) patients with T2DM compared to control subjects. We also found that serum adiponectin concentrations were significantly lower in T2DM compared to control subjects, in keeping with the current research literature [25]. Both cartonectin and adiponectin have apparently similar functions i.e. insulin sensitizing, anti-inflammatory and cardio-protective properties [26], and may function synergistically. Notably, Wolfing *et al*. had shown that cartonectin stimulates adipocyte adiponectin secretion [15]. Furthermore, we had observed significantly lower leptin concentrations in our newly diagnosed (first discovery) T2DM subjects (median HbA1c: 7.3%). This is similar with previous publications describing lower leptin concentrations in subjects with poorly controlled T2DM [27], [28].

The lower serum cartonectin concentrations in patients with T2DM is bewildering given that Choi *et al*. had recently reported that plasma cartonectin was significantly higher in patients with T2DM [29], and that a three month combined exercise program significantly decreased cartonectin levels in obese Korean women [30]. Interestingly, the same researchers also reported that serum cartonectin was not significantly lower in subjects with the metabolic syndrome compared to controls [31]. More recently, Choi *et al*. had published data reporting significantly lower circulating cartonectin concentrations in patients with acute coronary syndrome or stable angina pectoris, compared to control subjects [32]. A possible explanation of elevated cartonectin levels in T2DM observed by Choi *et al*. [29] could be the effect of medications taken by their study subjects as discussed in their following manuscript [31]. We have reported that metformin significantly increases circulating cartonectin concentrations in insulin resistant women with PCOS [33]. The findings from the current study support this notion.

Also, we present novel data that serum cartonectin concentrations were significantly lower in T2DM patients after a 2 hour 75 g OGTT. In contrast, glucose and insulin concentrations were significantly higher after the 2 hour 75 g OGTT. Therefore, glucose and/or insulin could account for the reduction in serum cartonectin concentrations. Since a recent *in vitro* study demonstrated that cartonectin concentrations were increased by insulin [34], and that glucose was significantly altered (lower) but not insulin in our T2DM patients, we propose that changes in glucose metabolism are more likely to explain our findings.

A limitation of this study is that it comprised of only Chinese subjects and thus our observations may not apply to other populations. Further studies are needed to elucidate this point. Notwithstanding, our results contribute to the paucity of human studies on cartonectin and raises interesting questions on the factors that regulate cartonectin concentrations in human subjects with metabolic and cardiovascular complications.

Our data supports cartonectin as a potential novel biomarker for the prediction and early diagnosis of T2DM patients. Furthermore, cartonectin and/or pharmacological agents that increase circulating cartonectin levels can represent a new therapeutic field in the treatment of T2DM patients. Further research is needed to clarify these points.
